# 2‐Chloroprocaine vs. Lidocaine in a Patient With Hypermobile Ehlers–Danlos Syndrome and a History of Local Anesthetic Resistance: A Case Report

**DOI:** 10.1155/cria/8693646

**Published:** 2026-04-10

**Authors:** Jayanth Dasika, Zachary B. Deutch, Amie L. Hoefnagel

**Affiliations:** ^1^ Department of Anesthesiology UF Health Jacksonville, University of Florida-Jacksonville, Jacksonville, Florida, USA, ufl.edu; ^2^ US Anesthesia Partners, Palm Coast, Florida, USA

## Abstract

Patients with hypermobile Ehlers–Danlos syndrome (hEDS) have a higher reported prevalence of local anesthetic (LA) resistance and obstetric complications than the general population. We describe a 33‐year‐old G2P0010 ASA III parturient with a history of hEDS and lidocaine resistance who presented for an anesthesia consultation in anticipation of possible neuraxial anesthesia. We surmised that performing a neuraxial procedure would require effective skin infiltration of the patient’s lumbar area. A skin infiltration test revealed that 3% 2‐chloroprocaine provided a much greater numbing effect than 2% lidocaine. The patient desired an unmedicated birth and ultimately delivered in this fashion. Still, our findings highlight the potential benefit of testing multiple LAs in hEDS patients to guide anesthetic planning.

## 1. Introduction

Ehlers–Danlos Syndrome (EDS) is a group of rare genetic connective tissue disorders characterized by joint hypermobility, skin hyperextensibility, and tissue fragility. Anesthetic considerations were first described in 1980, including the potential complications of wound dehiscence and increased bleeding [1]. Local anesthetic (LA) resistance was subsequently described in a smaller study of EMLA cream application in 8 hEDS patients, which found reduced cutaneous analgesia compared to controls. In another study, infiltration with 1% lidocaine did not provide sufficient analgesia [2]. A larger, more recent survey found a high prevalence of LA resistance, with EDS survey respondents reporting nearly three times the rate of LA nonresponse compared to non‐EDS respondents [3].

Regional and neuraxial anesthetics have been described in hEDS, with several case reports of success using either technique [4–6]. One case report involved inadequate pain relief despite successful placement of an epidural catheter for a laboring parturient. The patient felt pain on insertion of the Tuohy needle and later required frequent top‐ups of the maintenance infusion (0.1% bupivacaine with fentanyl 2 μg/mL). She eventually used nitrous oxide for supplemental analgesia. Upon later questioning, the patient admitted to undergoing minor procedures under local anesthesia in the past and experiencing significant discomfort [7].

Patients with hEDS are prone to a higher rate of obstetric complications including rapid labor, hemorrhage, poor wound healing, pelvic floor complications, and psychological morbidity [8]. These risks highlight the importance of multidisciplinary planning and heightened surveillance during the peripartum period.

Informed consent was obtained from the patient for the publication of this case report.

## 2. Case Presentation

A 33‐year‐old G2P0010 with a past medical history of hEDS, anxiety, depression, and migraines presented at 41w1d gestational age, in latent labor. Although the patient preferred an unmedicated birth, an anesthetic consult was requested due to the high‐risk obstetric nature of hEDS, combined with the patient’s personal history of LA resistance, specifically to lidocaine during a dental procedure.

The patient was diagnosed with hEDS at Age 31. She then underwent pharmacogenomic testing, which indicated LA hypermetabolism. Results of OneOme testing revealed the following relevant cytochrome P450 phenotypes: rapid metabolism of CYP1A2 and mildly reduced to normal metabolism of CYP3A4. This likely explained lidocaine’s ineffectiveness as it is primarily metabolized by CYP1A2. It also would predict mostly normal metabolism of bupivacaine due to a normal CYP3A4 phenotype.

The patient agreed to the following plan for her delivery: An elective labor epidural could be placed (if needed) if an alternative to lidocaine for skin infiltration could be found. The epidural infusion itself, whether LA combined with opioid, or either medication alone, could be selected on a trial‐and‐error basis. If an urgent C‐section was needed, neuraxial anesthesia would be attempted and, if it could not produce a surgical block, general anesthesia (GA) would be initiated. GA would also be the default for an emergent/stat C‐section. Large‐bore IV access would be attempted, and uterotonic medications including oxytocin, methylergonovine, and prostaglandin would be available to mitigate excessive hemorrhage. The patient would be typed and cross‐matched for blood products.

As mentioned above, in order to facilitate a potential neuraxial procedure, an alternative to lidocaine would be needed for skin infiltration. On this basis, the patient agreed to be tested with a sample of LA to see which, if any, might be effective. 2% lidocaine and 3% 2‐chloroprocaine were tested by 1 mL subcutaneous infiltration in the left and right forearm, respectively, and assessing skin sensation. The patient was blinded to the anesthetic injected. Pain response was tested after 1 min via pinprick using an 18‐gauge blunt‐fill needle. Lidocaine demonstrated mild reduction in sensation to pinprick, whereas 2‐chloroprocaine demonstrated a marked reduction in sensation. As per her preference, the patient was ultimately able to forgo neuraxial (or other) analgesic interventions and experienced an unmedicated and uncomplicated vaginal delivery.

## 3. Discussion

Our patient’s history of inadequate anesthesia during a dental procedure aligns with many hEDS patients reporting a similar outcome. Out of 980 dental patients surveyed with EDS, 88% recalled inadequate analgesia after LA injections—nearly three times the rate of those without EDS [3]. As in this case, lidocaine seemed to be a common culprit for providing poor efficacy. Of those respondents receiving lidocaine, only 8% indicated feeling any effect. In another study, infiltration with 1% lidocaine provided insufficient analgesia compared to controls [2]. Honore et al. found a higher rate of LA insufficiency, although there is no mention of a specific LA [9].

Being an ester‐type LA, primarily metabolized by pseudocholinesterase, 2‐chloroprocaine would likely have provided more effective analgesia for our patient. The medication is not affected by genetic variations in cytochrome p450 that can affect amide‐type LAs such as lidocaine.

Although bupivacaine is commonly used for neuraxial anesthesia, we elected not to test it in the patient due to the rapid onset of 2‐chloroprocaine and the ability to see the empirical results of our skin testing within minutes. The use of bupivacaine in this patient would likely have been efficacious, given that her pharmacogenomic testing indicated normal metabolism.

Inherited variations or point mutations of voltage‐gated sodium channels could alter drug binding sites or downstream signaling [10]. Factors related to abnormal connective tissue (i.e., skin laxity) may be involved but are not fully understood. Dispersion rates of LA through deep dermal injections were identical for both EDS patients and control subjects [11]. Other mechanisms have been hypothesized but none are specific to EDS, nor have they been tested.

The existing literature is limited by its qualitative and subjective nature, primarily depending on surveys and patient experience for interpretation of pain. What is clear from our results, however, is that some LAs may work in hEDS patients when others do not. Patients should be counseled about this, and neuraxial procedures are often a viable option.

This case underscores the variability of LA efficacy in hEDS and the importance of individualized testing, particularly when labor analgesia or surgical anesthesia is needed.

## Funding

No funding was received for this manuscript.

## Conflicts of Interest

The authors declare no conflicts of interest.

## Data Availability

Data sharing is not applicable to this article as no datasets were generated or analyzed during the current study.
